# Safety enhancement of improved hydrodissection for microwave ablation in lymph node metastasis from papillary thyroid carcinoma: a comparative study

**DOI:** 10.3389/fendo.2025.1594561

**Published:** 2025-07-23

**Authors:** Jie Wu, Ying Wei, Zhen-long Zhao, Shi-liang Cao, Yan Li, Li-li Peng, Shu-qi Li, Ming-an Yu

**Affiliations:** Department of Interventional Medicine, China-Japan Friendship Hospital, Beijing, China

**Keywords:** lymph node metastasis, papillary thyroid cancer, hydrodissection, microwave ablation, fascial space

## Abstract

**Purpose:**

This study aims to evaluate the efficacy and safety of an improved hydrodissection technique based on the perilymph-nodal space (PLNS) when applied during microwave ablation for treating lymph node metastases (LNM) arising from papillary thyroid carcinoma (PTC).

**Methods:**

A retrospective analysis was conducted on data from 266 patients (95 males, 171 females, mean age 41.3 ± 14.0 years, range 16–88) who underwent MWA for LNM. Of these, 142 patients received traditional hydrodissection (traditional group), while 124 underwent the improved technique. Safety outcomes were assessed by comparing complication rates between the two groups. Additionally, the characteristics of the hydrodissected fascial spaces, complications, and follow-up results were documented.

**Results:**

All patients underwent successful hydrodissection as planned. The improved hydrodissection group demonstrated a lower incidence of hoarseness compared to the traditional group (4.8% vs. 8.4%, p >0.05). Notably, in region VI cases, the improved technique significantly reduced the incidence of hoarseness (7.5% vs. 25%, p = 0.006). Additionally, the median recovery time for hoarseness was shorter in the improved group (3 vs. 6 months, p <0.05). During follow-up, neither group exhibited local recurrence. The tumor disappearance rates were comparable between the groups (75.4% vs. 65.3%, p >0.05).

**Conclusion:**

The PLNS-based improved hydrodissection technique demonstrated enhanced safety compared to traditional hydrodissection during MWA for LNM, especially for region VI lesions.

## Background

Papillary thyroid carcinoma (PTC) is the most common thyroid cancer, accounting for the majority of cases. While most cases are successfully managed through surgery or ablation, approximately 15%–30% of patients experience local recurrence and cervical lymph node metastasis (LNM) (1–3). According to guidelines from the American Thyroid Association, surgical removal is the recommended approach for managing neck LNM, despite the inherent risks of reoperation (4). Postoperative fibrosis may alter tissue adhesions and disrupt anatomical structures, complicating subsequent surgeries. Repeated procedures may increase the incidence of both transient and permanent nerve complications. Additionally, some patients face surgical challenges due to underlying health conditions or may choose to forgo surgery. Therefore, identifying a non-surgical, minimally invasive approach for managing LNM is critically important. Recently, ultrasound (US)-guided thermal ablation techniques, including microwave ablation (MWA) and radiofrequency ablation (RFA), have been introduced in clinical practice as less invasive treatments for LNM, offering several advantages (5–11).

The hydrodissection technique serves as a protective strategy to minimize thermal injury to surrounding structures during ablation and has been recommended in various guidelines for thyroid tumors (12, 13). This fascial space-based approach has also been similarly applied in the thermal ablation of LNM (6). However, no comprehensive study has yet examined hydrodissection as a standard procedure for LNM ablation.

Based on clinical experience with microwave ablation (MWA) for more than 700 LNM, we developed an improved hydrodissection protocol at our center, focusing on perilymph-nodal spaces. In the present study, we describe this improved hydrodissection protocol and evaluate its safety enhancements by comparing it with traditional hydrodissection techniques used in MWA for LNM.

## Materials and methods

### Study design and patients

This retrospective cohort study was approved by the Human Ethics Review Committee of the China–Japan Friendship Hospital. Written informed consent was obtained from all patients prior to ablation. Additional consent for research participation was waived, as the study used anonymized clinical records.

Medical records were reviewed for all consecutive patients with lymph node metastasis (LNM) who underwent microwave ablation (MWA) from November 2015 to September 2024 at our center. Inclusion criteria were: (1) confirmation of LNM from papillary thyroid carcinoma (PTC) via fine needle aspiration (FNA) before ablation; (2) refusal of neck dissection; (3) follow-up duration exceeding 3 months; (4) maximum LNM diameter ≤5 cm; and (5) absence of severe adhesion or invasion into adjacent structures. Exclusion criteria included: (1) patients under 16 years old or pregnant; (2) presence of distant metastasis; (3) history of more than three times of surgical resections; (4) Peri-LNM adhesion resulting in inability for hydrodissection; and (5) serious bleeding tendencies.

A total of 266 patients with 735 LNM lesions were enrolled, comprising 95 males and 171 females (median lesion volume: 0.919 mL [interquartile range (IQR): 0.471 mL–2.300 mL]). The mean age was 41.3 ± 14.0 years (range: 16–88 years). Patients were stratified into two cohorts based on the hydrodissection technique used: the traditional group and the improved group. Traditional hydrodissection, generally based on the fascial spaces surrounding LNM, was performed from November 2015 to December 2022. Based on accumulated clinical experience, an improved hydrodissection technique focusing on the LNM capsule has been implemented since January 2023.

### Equipment

Ultrasound (US) examination and microwave ablation (MWA) guidance were performed using either (1) the GE LOGIQ E9 system (GE Healthcare, Pittsburgh, PA, USA) equipped with a 9.0-MHz linear-array transducer, or (2) the Aplio 500 system (Toshiba, Tokyo, Japan) featuring a 10.0-MHz linear-array transducer. For MWA, a 17-G internally cooled antenna with a 3-mm active tip (Nanjing ECO Microwave System) was used. For CEUS, either SonoVue (Bracco, Milan, Italy) or Sonazoid (Daiichi-Sankyo, Tokyo, Japan) was used as the ultrasound contrast agent. Hydrodissection and subsequent ablation procedures were performed by three radiologists, each with 5 years of experience in ultrasound for lymph node metastasis (LNM).

### Traditional and improved hydrodissection procedures

Patients were positioned supine with the neck extended. The designated ablation area was thoroughly disinfected and then draped using sterile technique. A subcutaneous injection of 1% lidocaine was administered at the puncture site. Subsequently, an 18 G-21G core needle connected to an extension tube and filled with normal saline (NS) was inserted layer by layer under ultrasound guidance. Once the needle tip reached the target area, NS was gently injected.

**Figure 1:**
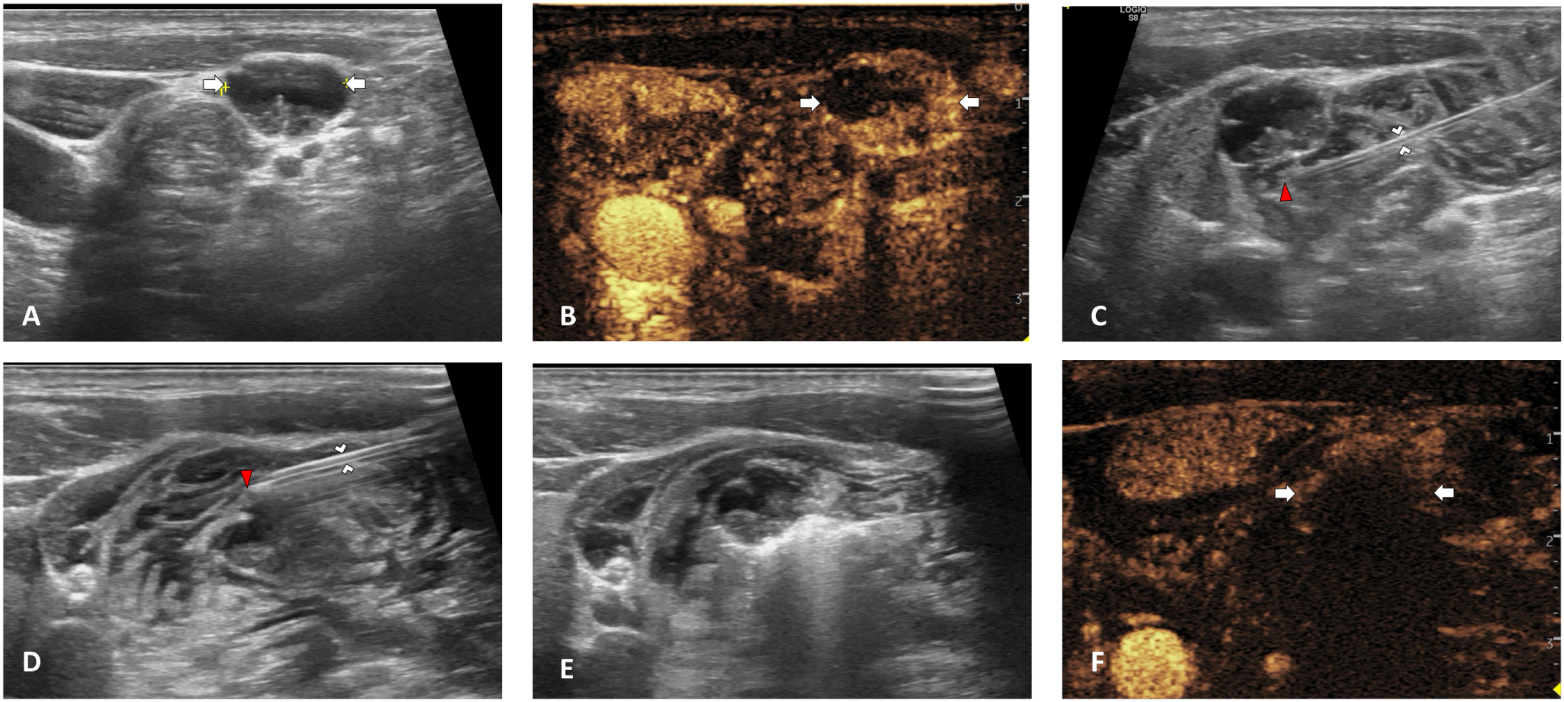
Ultrasound images of traditional hydrodissection. **(A)** Pre-MWA, B-mode ultrasonography (US) showed hypoechoic LN (white arrow). **(B)** Pre-MWA, Contrast-enhanced ultrasound (CEUS) showed inhomogeneous enhanced LN (white arrow). **(C)** Traditional hydrodissection before ablation. The needle tip (red arrowhead) was placed in the posterior fascial spaces outside the LNM capsule and forming a narrow anechoic isolating band outside the LNM lesion in the posterior space. **(D)** Traditional hydrodissection before ablation. The needle tip (red arrowhead) was placed in the anterior fascial spaces outside the LNM capsule and forming a narrow anechoic isolating band outside the LNM lesion in the anterior space. **(E)** During ablation, the thickness of isolating band was maintained through continuous injection of NS. **(F)** Post-MWA, CEUS showed no enhancement in LN (white arrows).

**Figure 2:**
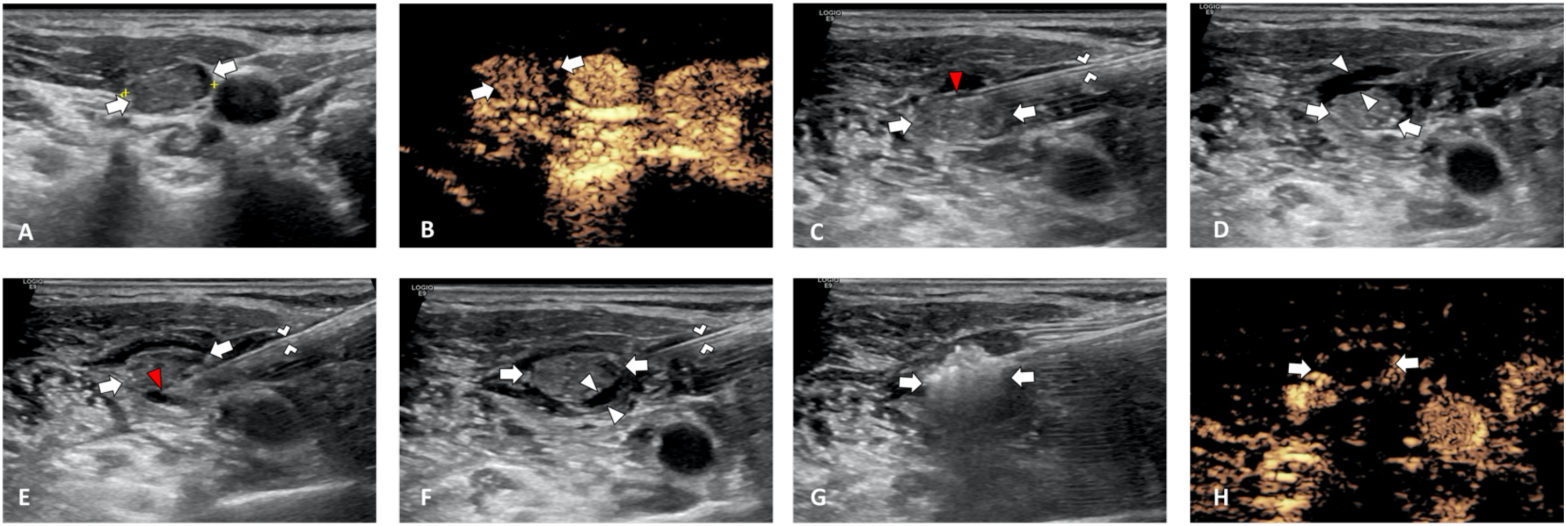
Ultrasound images of improved hydrodissection. **(A)** Pre-MWA, B-mode ultrasonography (US) showed hypoechoic LN (white arrow). **(B)** Pre-MWA, Contrast-enhanced ultrasound (CEUS) showed inhomogeneous enhanced LN (white arrow). **(C)** Improved hydrodissection before ablation. The needle tip (red arrowhead) was inserted and fixed inside the anterior LNM capsule. **(D)** A semi-circular anechoic isolating band (white arrowhead) was formed outside the LNM lesion (white arrow) in the anterior space. **(E)** Improved hydrodissection before ablation. The needle tip (red arrowhead) was inserted and fixed inside the posterior LNM capsule. **(F)** A semi-circular anechoic isolating band (white arrowhead) was formed outside the LNM lesion (white arrow) and forming perilymph-nodal space (PLNS). **(G)** During ablation, the heat energy was kept inside the LNM lesion (white arrow). **(H)** Post-MWA, CEUS showed no enhancement in LN (white arrowhead).

**Figure 3:**
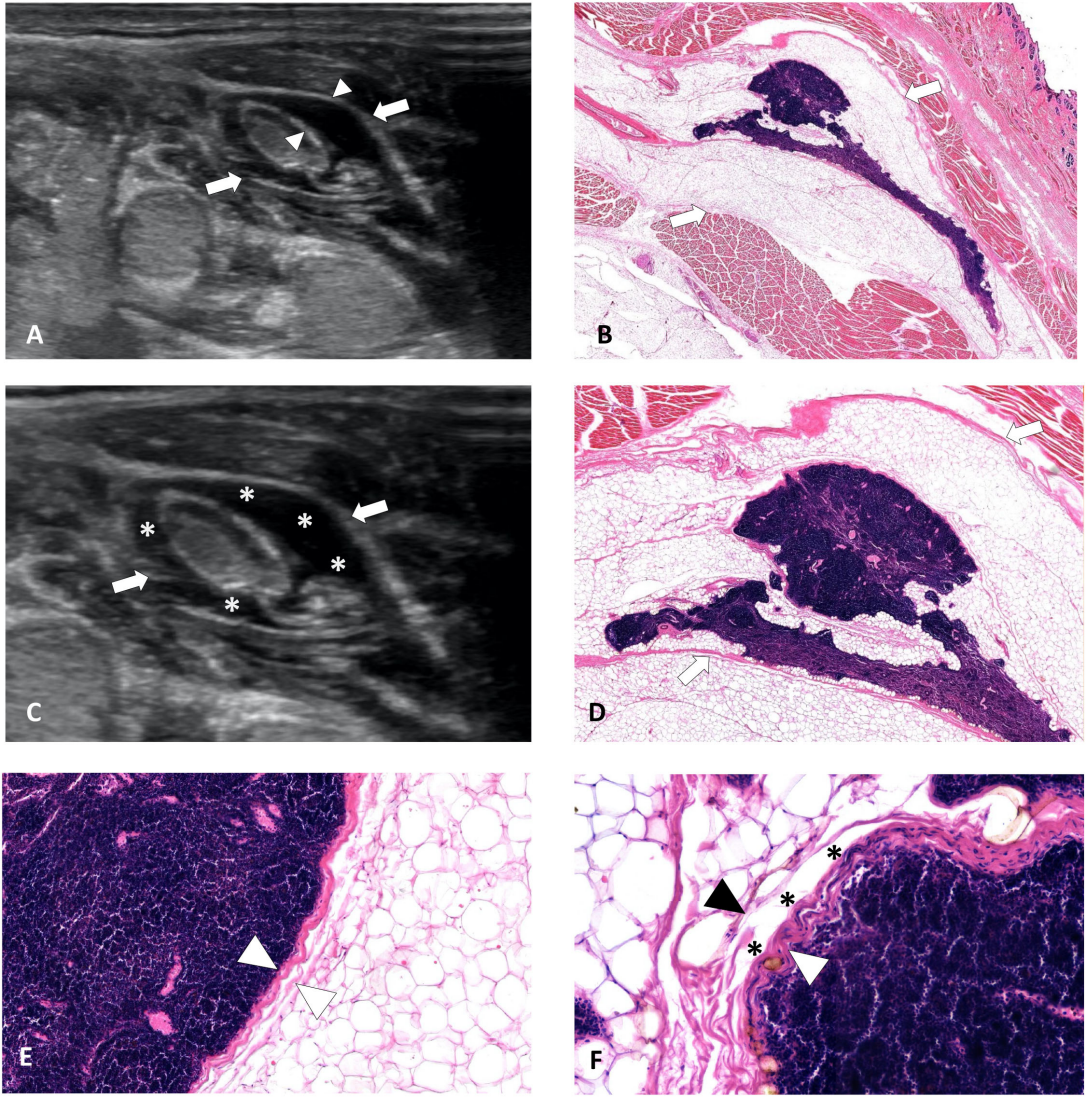
Ultrasound image of hydrodissected PLNS and the pathological images. **(A)** Ultrasound image shows fascial space (white arrows) between LN and surrounding muscles. The PLNS (white arrowhead) shows as an anechoic or mixed-echoic isolating band after hydrodissection with a clear and smooth border and tension due to restricted fluid. **(B)** Pathological image from rabbit shows corresponding fascia (white arrows) between LN and surrounding muscles. **(C)** The PLNS (asterisks, *) shows as the ‘onion skin’ sign, an annular multilayer anechoic area with a distinct border and tension surrounding the LN lesion. **(D, E)** The MASSON stains of resected LN lesion from rabbit showed there were several layers of circular distributed collagen fibers (white arrows and white arrowheads) around LN lesion. **(F)** Pathological image from rabbit shows that the PLNS (asterisks, *) is a potential gap between LN capsule (white arrowhead) and surrounding fat tissue (black arrowhead). Note: PLNS, perilymph-nodal space.

For traditional hydrodissection, the 18 G-21G core needle tip was placed in the fascial spaces outside the LNM capsule, and NS was injected to form an anechoic zone within the fascial spaces, creating a distance of at least 5 mm between the LNM and critical structures (**Figure 1**). During injection, the fluid flowed along the fascial spaces. For improved hydrodissection, the needle tip was initially inserted and fixed within the LNM capsule. NS was injected, forming an annular anechoic region within the LNM capsule and creating the perilymph-nodal space (PLNS) (**Figure 2**). During injection, the liquid diffused from the PLNS to outside the capsule, reaching the fascial spaces surrounding the LNM. The needle tip was securely fixed within the capsule of the LNM. During ablation, NS was continuously injected as the isolating fluid to maintain an isolating band of at least 5 mm width, giving the LNM an ‘island-like’ appearance (**Figure 3**). The PLNS was also validated by comparing US imaging with microscopic pathology in this study (**Figure 3**).

Ultrasound examination of following successful hydrodissection reveals characteristic fascial space features, including: (1) well-defined, smooth margins; (2) tissue tension due to constrained fluid distribution; (3) formation of a protective fluid barrier (anechoic or of mixed echogenicity) that displaces adjacent critical structures from the LNM; and (4) the distinctive “onion skin” sign—a multilayered anechoic region with sharp demarcation and circumferential tension around the LNM (**Figure 3**).

### Preablation assessment and MWA procedure

Pretreatment evaluation and microwave ablation (MWA) were performed according to established protocols (5, 6). All LNMs identified by ultrasonography (US) were targeted for complete ablation within a single session. However, in cases where the LNMs were excessively large or numerous, or when unilateral recurrent laryngeal nerve (RLN) injury occurred, ablation of contralateral LNMs was postponed, requiring a second-session ablation protocol.

### Postablation assessment and follow-up visit

Complete ablation was defined as the presence of a non-enhanced ablation zone entirely covering the LNM on contrast-enhanced US. Technical success was defined as complete ablation achieved according to the established protocol. After ablation, all patients were scheduled for a first follow-up at 1 month, followed by follow-ups every 3 months during the first year and 6 months thereafter. Local recurrence was defined as tumor regrowth along the margin of the LNM ablation zone observed on US during follow-up examinations. LNM disappearance was defined as complete absorption of the ablated target LNM on US. Follow-up assessments included cervical lymph node US and computed tomography (CT). Additionally, in patients presenting with hoarseness, vocal cord movement was evaluated via US at each follow-up visit.

### Statistical methods

All statistical analyses were performed using SPSS Statistics (version 24.0; IBM Corp., Armonk, NY). Continuous variables with normal distribution were expressed as mean ± standard deviation (SD), while non-normally distributed variables were reported as median with interquartile range (IQR; 25th–75th percentiles). Comparative analyses were conducted using appropriate statistical tests: independent samples t-test for normally distributed continuous variables, Mann–Whitney U test for nonparametric data, and chi-square test for categorical variables. To identify potential risk factors for hoarseness, logistic regression with forward stepwise variable selection was used. All statistical tests were two-tailed, and a p-value <0.05 was considered statistically significant.

## Results

### Patient characteristics

Patients were stratified into two treatment subgroups: 142 patients (453 lesions) received traditional hydrodissection, while 124 patients (284 lesions) underwent improved hydrodissection. Comparative analysis showed balanced baseline parameters between the two groups, with no statistically significant differences in demographic characteristics (sex, age) or LNM lesion morphology (size, volume) (all p >0.05). Detailed patient demographics and clinical characteristics are presented in **Table 1**.

**Table 1:** Clinical characteristics of the study population.

Variables	Traditional hydrodissection group (n = 142)	Improved hydrodissection group (n = 124)	*p* Value
Age (years)*	42.30 ± 14.0	40.02 ± 13.66	0.397
Gender (M:F)	46:96	49:75	0.227
Maximum diameter (cm)^a^	1.0 (0.8–1.5)	1.0 (0.73–1.5)	0.560
	Range: 0.3–4.8	Range: 0.2–3.9	
Tumor location (n of LNM))	453	284	/
Region VI	169	121	0.15
Non-region VI	284	163	
Total hoarseness rate	12/142	6/124	0.242
Hoarseness rate in Region VI	12/48	6/80	0.007**
Hoarseness recovery time (month)^a^	6 (3–6)	3 (1.75–3)	0.037**

*Data presented as mean ± standard deviation (range). ^a^Data are presented as the median (25%–75% interquartile range). **p <0.05.

### Hydrodissection outcomes

#### General information on hydrodissection

**Figure 4:**
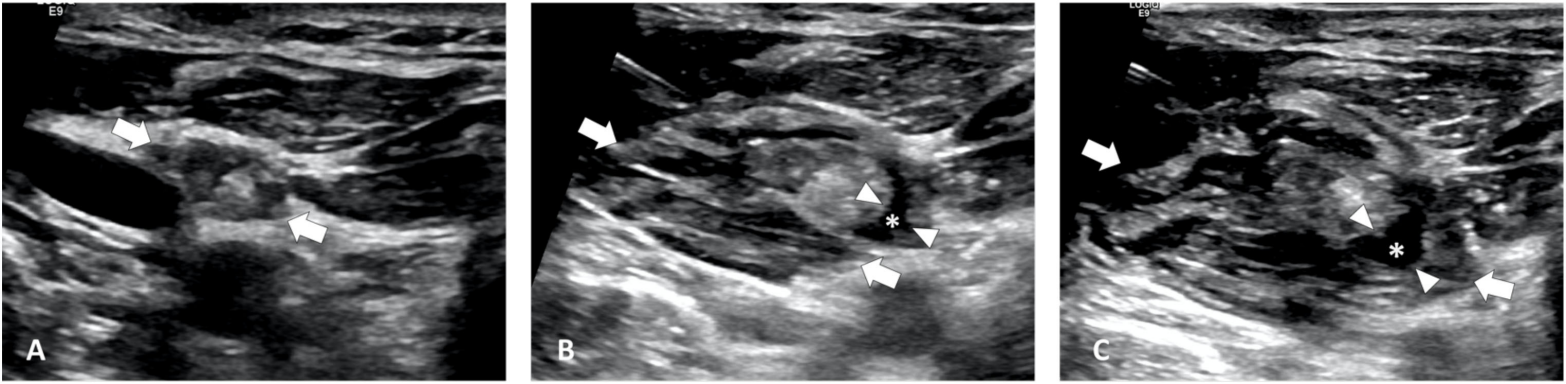
Ultrasound Image of continues hydrodissection. **(A)** Pre-MWA, B-mode ultrasonography (US) showed hypoechoic LN (white arrows). **(B, C)** Under continues injection, the NS solution inside PLNS (asterisks, * between white arrowheads) diffuse to outside of LNM capsule to surrounding spaces (white arrows), further pushing important structures away.

In this study, the perilymph-nodal space (PLNS) was first identified via ultrasound (US) during hydrodissection. The PLNS was defined as the region surrounding the LNM, composed of multilayered, continuous collagenous fibers forming an onion-skin-like structure with distinct boundaries and circumferential tension after hydrodissection, isolating the LNM from adjacent critical structures. The PLNS was hydrodissected only in the improved hydrodissection, and not in the traditional technique. A schematic representation of the hydrodissection space is shown in **Figure 4**.

#### Ablation outcome

Hydrodissection (both traditional and improved techniques) was successfully completed in all cases according to protocol, achieving a technical success rate of 100%.

Post-ablation CEUS confirmed complete absence of enhancement in all targeted LNMs, with identical 100% technical success rates observed in both groups. The ablation power ranged from 30 W to 40 W in both the traditional and improved groups. The overall median ablation time was 36.0 s per lesion [interquartile range (IQR): 20.0 s–67.0 s]. Although the improved group showed a shorter median time (30.0 s) compared to the traditional group (39.8 s), the intergroup difference was statistically non-significant (p >0.05). Moreover, no significant difference was found between the two groups in total procedure duration, including both ablation and hydrodissection. RLN injury-induced hoarseness was the only observed major complication, occurring in 18 total cases: six cases (4.8%, 6/124) in the improved group and 12 (8.4%, 12/142) in the traditional group (p >0.05). All cases of hoarseness occurred exclusively in patients with level VI LNM. The improved hydrodissection technique significantly reduced the incidence of hoarseness in level VI LNMs compared to the traditional group (7.5%, 6/80 vs. 25%, 12/48; p = 0.006). Permanent hoarseness was observed in one patient in the traditional group and in none of the improved group, although the difference was not statistically significant (p >0.05). Among 17 transient hoarseness cases, all patients achieved full vocal recovery, with a significantly shorter median recovery time in the improved hydrodissection group (3 months vs. 6 months, p <0.05).

Follow-up observations revealed no local tumor recurrence in either group. At the final follow-up, complete tumor disappearance was observed in 75.4% (107/142) of cases in the traditional group and 65.3% (81/124) in the improved group, with no statistically significant difference (p >0.05).

## Discussion

Over the past decade, US-guided MWA has been preliminarily applied in clinical practice for managing LNM from PTC, achieving promising results (5–11). Nevertheless, LNM are frequently adjacent to vital structures, including the trachea, esophagus, and nerves. Moreover, delicate nerves remain undetectable by US in most clinical scenarios, particularly the superior laryngeal nerves and RLNs, which are highly susceptible to thermal injury. The incidence of RLN injury of MWA and RFA for LNM has been reported to be as high as 0.5%–0.7% in previous studies (14). Thus, maintaining procedural safety remains a crucial yet challenging aspect of MWA for LNM.

Hydrodissection is a crucial technique for minimizing heat injury by creating a safety band between the LNM and surrounding vital structures during ablation. Wei et al. reported ultrasound imaging of the peri-thyroid fascial space, demonstrating that US-guided hydrodissection facilitates fascial space visualization (15). Zhao et al. reported an improved hydrodissection strategy based on fascial spaces for thermal ablation of thyroid tumors, which demonstrated enhanced protection of critical structures during ablation (16). However, this technique remains an empirical approach in LNM ablation, with limited studies establishing it as a standardized procedure. In the present study, the PLNS was first identified by US during hydrodissection, and an improved hydrodissection technique based on PLNS was also first established. The technical success rate of hydrodissection was 100% in both the traditional and improved groups, demonstrating the feasibility of the improved PLNS-based technique.

According to follow-up results, improved hydrodissection significantly enhanced the safety of ablation, particularly for LNM lesions in region VI—an anatomically challenging location. The incidence of RLN injury was lower in the improved group (4.8% vs. 8.4%), and significantly lower in the region VI subgroup (7.5% vs. 25.0%). These findings suggest that the improved hydrodissection technique can effectively reduce the incidence of RLN thermal injury during MWA of LNM in region VI. LNMs in region VI region are adjacent to the recurrent laryngeal nerve (RLN), which is not visible on ultrasound. Traditional hydrodissection only separates the space surrounding the LNM, where the RLN may also be located, making effective separation between the RLN and LNM difficult to ensure. The improved hydrodissection based on the PLNS effectively separates the RLN from the LNM, resulting in a statistically significant reduction in RLN injury between the two groups. For LNMs in the lateral neck, the RLN is generally absent, and the incidence of hoarseness was 0% in both groups, resulting in no statistical difference. Additionally, compared with traditional hydrodissection, the improved technique yielded both faster recovery (3-month vs. 6-month convalescence) and no cases of permanent voice impairment, clearly demonstrating its safety advantages. Furthermore, the incidence of RLN injury in the improved group was lower than the 19.7% reported after central neck dissection (CND) (17), and the 6.25% reported after repeated lymph node dissection (7), further confirming its safety advantage (**Table 1**).

The reasons why the improved hydrodissection technique may enhance the safety of ablation are as follows: (1) the improvement hydrodissection based on PLNS may effectively prevent heat spillover beyond the LNM capsule; (2) maintaining a separation distance of at least 5 mm may help displace critical structures away from LNM; (3) continuous injection of isolation fluid can effectively maintain a sufficient separation distance; (4) the NS solution within the PLNS may diffuse beyond the LNM capsule into surrounding spaces during continues injection, further displacing critical structures (**Figure 4**); (5) furthermore, in cases of postoperative adhesions in the surrounding fascial spaces, the PLNS could still be successfully hydrodissected in most situations, potentially increasing the likelihood of safe and effective LNM ablation using PLNS-based hydrodissection.

Several methodological limitations should be acknowledged. First, the sonographic assessment of fascial spaces lacked histopathological correlation with gross anatomical specimens. Second, the retrospective study design carries inherent limitations, including potential selection and information biases.

## Conclusions

The development of this PLNS-based improved hydrodissection technique may enhance ablation safety and facilitate the broader clinical adoption of thermal ablation as a minimally invasive treatment for LNM.

## Data Availability

The original contributions presented in the study are included in the article/supplementary material. Further inquiries can be directed to the corresponding author.
